# The Influences of Cement Hydration and Temperature on the Thixotropy of Cement Paste

**DOI:** 10.3390/ma13081853

**Published:** 2020-04-15

**Authors:** Julian Link, Thomas Sowoidnich, Christopher Pfitzner, Teba Gil-Diaz, Frank Heberling, Johannes Lützenkirchen, Thorsten Schäfer, Horst-Michael Ludwig, Michael Haist

**Affiliations:** 1 Institute for Building Materials Science, Leibniz University Hannover, Appelstraße 9a, 30167 Hannover, Germany; j.link@baustoff.uni-hannover.de (J.L.); chris.pfitzner@posteo.de (C.P.); 2 F.A. Finger-Insitute for Building Materials, Bauhaus University Weimar, Coudraystrasse 11, 99423 Weimar, Germany; thomas.sowoidnich@uni-weimar.de (T.S.); horst-michael.ludwig@uni-weimar.de (H.-M.L.); 3 Karlsruhe Institute of Technology (KIT), Hermann-von-Helmholtz-Platz 1, 76344 Eggenstein-Leopoldshafen, Germany; teba.gil-diaz@uni-jena.de (T.G.-D.); frank.heberling@kit.edu (F.H.); Johannes.luetzenkirchen@kit.edu (J.L.); 4 Institute of Geosciences, Friedrich-Schiller-Universität Jena, Burgweg 11, 07749 Jena, Germany; thorsten.schaefer@uni-jena.de

**Keywords:** rheology, hydration, thixotropy, cement paste, opus fluidum futurum

## Abstract

The rheological properties of fresh cement paste are highly influenced by a large number of parameters, among which the most important factors are the applied shear stress, and the shear history, the age of the sample and the temperature. The effects of these parameters on the yield stress (designated as structural limit stress in this work), the viscosity and the structural recovery rate (i.e., the change in dynamic viscosity with time at rest) were studied. In parallel, the changes in ion composition of the carrier liquid, mineral phase content and granulometry were investigated. The results reveal that all investigated rheological parameters exhibit an approximated bi-linear trend with respect to the degree of hydration, with a period of quasi-constant properties until a degree of hydration of approximately 0.07, followed by a non-linear increase. This increase could be attributed to the formation of calcium hydroxide (CH) and calcium-silicate-hydrate (C-S-H) via calorimetry results. With regard to the effect of the shear history of the sample on the rheological properties, the structural limit stress showed a minor dependency on the shear history immediately after the end of shearing, which, however, vanished within the first minute at rest. The same is true for the structural recovery rate. The presented results give detailed insights into the influences of hydration and shear on the rheological properties—especially the thixotropy—of fresh cement pastes.

## 1. Introduction

The control of the rheological properties of fresh concrete during casting is essential for the production of high-quality concrete structures. The concrete consistency must be adjusted to ensure sufficient flow in order to completely fill the formwork, and good compactibility for the enclosed air to escape the concrete during the compaction process.

The rheological properties of fresh concrete are subject to a great variety of influencing factors. Among the most important are the age of the concrete (time after water addition), the shear history—i.e., the shear the concrete was previously exposed to, e.g., due to mixing, transport and placement—and the temperature (see, e.g., [1]). All of these influencing factors primarily act on the interaction behavior of the fine particles—i.e., the cement. The investigations in this paper are thus carried out on fresh cement paste, which allows for a much more detailed study of the underlying processes.

In order to predict the rheological properties of fresh cement paste, mortar or concrete, models based on the DLVO-theory by Derjaguin, Landau, Vervey and Overbeek (see, e.g., [2]) are very common. The DLVO-theory in principle can predict the agglomeration state of cement particles in suspension. These particle-interaction-based schemes may be coupled with semi-empirical models to predict the rheological properties, normally expressed by the Bingham yield stress *τ*_0_ and the Bingham plastic viscosity μ (see e.g., [3,4,5]). A key deficit of such models is the fact that they idealize cement as quasi inert, chemically homogeneous material with spherical particles and thus do not capture the time-dependent change in particle inventory due to hydration nor physical effects such as time-dependent agglomeration. Both processes, however, are highly interactive. With progressing hydration, especially very fine cement particles dissolve quickly [6], which goes along with a significant increase in ionic strength. The high ion strength of the aqueous phase, e.g., up to 1.0 M—which acts as a carrier liquid for the cement particles—causes a very low Debye-length of the diffuse layer that surrounds the cement particles [2,6,7]. As a consequence, the inter-particle forces in cement pastes are mainly attractive (van der Waals), causing agglomeration. Second, the increase in ionic strength due to continuous dissolution results in saturation of the carrier liquid with ions, leading to the formation of new mineral phases, which act in a steric manner [8,9,10].

The influences of these dissolution and precipitation processes—especially the temporal evolution of these processes—on the rheological properties of cement paste, are not understood. As a consequence, key material properties, such as the age dependent stiffening of the paste due to early hydration of the cement, and thixotropy, i.e., the change in dynamic viscosity *η* at rest or at constant shear, so far cannot be accurately predicted. This is especially true when superplasticizers are additionally used in the concrete production process.

In light of this background, substantial efforts have been made in recent years in order to investigate and model the origins of thixotropy in fresh cement paste and its dependence on the hydration of cement particles. Roussel and coworkers, for example, reasoned that thixotropy cannot be explained by a DLVO-driven particle agglomeration on its own but rather depends on the deposition of nano-scale calcium-silicate-hydrate (C-S-H) precipitates on the particles surfaces, leading to a mechanical bridging effect [11,12]. Own results show that, especially for later ages (i.e., beyond approximately 2 to 3 h after water addition) and higher temperatures (>30 °C), the formation of C-S-H significantly contributes to the stiffening of cement suspensions [13], supporting earlier findings on the role of alite (C_3_S) hydration on stiffening of fresh cement pastes [14,15]. Additionally, the hydration of tricalcium aluminate (C_3_A) is known to significantly influence the rheology of fresh concrete [16]. Jakob et al. relate the time dependent stiffening of fresh cement paste to the formation of ettringite on particle surfaces and found a good correlation between the amount of ettringite and the change in a viscosity parameter of the paste [17]. This finding is in line with results by Uchikawa et al. [18], who stated that the rheological properties of fresh cement pastes are governed in the early hydration stages by the formation of ettringite and later by C-S-H formation. Additionally, the formation of syngenite impacts the shear behavior of fresh cement pastes [19].

When looking at the results, close attention must be paid to the fact that rheological properties are measured and compared. Whereas in the work by Roussel [11,12] and by the authors of [3,13], the change in paste yield stress or stiffness was considered, the work of Rößler et al. and that of Jakob et al. addressed the change in the viscosity of the paste at somewhat higher shear rates [17,19]. Currently, it is not known how the mineralogical changes occurring during hydration influence the rheology, especially the thixotropy of the paste at low shear. The focus of the present research is thus directed at studying the changes in yield stress and viscosity at different temperatures at rest after a defined shear loading. The goal is to understand how and why structure is formed in cement paste and how this affects yield stress and viscosity. The term structure is hereby understood to describe the interactions of the solids in the paste, which allows for the transmission of forces without major viscous deformations having to occur.

In order to investigate this matter, fresh cement pastes were subjected to pronounced shear stresses for a defined duration, after which the shearing was stopped and the restructuring of the paste was monitored at different sample ages and temperatures. In parallel, the changes in chemical inventory and particle size distribution (not shown here) due to hydration were monitored for the same parameters in order to relate the changes in rheology to hydration processes.

## 2. Materials and Methods

### 2.1. Raw Materials and Sample Preparation

The cement used in the present investigation was Portland cement CEM I 42.5 R, in accordance with EN 197-1 [20]. The density of the cement was determined as 3.12 g/cm³ and the specific surface according to Blaine was 3600 cm²/g. The analysis of the particle size distribution yielded a mean particle size of 14.8 µm. A detailed physical analysis and the chemical and mineralogical composition are available in [21]. All cement paste samples were prepared with demineralized water. The temperature of the cement suspension was adjusted to 10, 20 or 30 °C, respectively, by pre-tempering the mixing water and the dry cement. For selected tests, a superplasticizer (SP) based on polycarboxylic-ether (PCE; experimental superplasticizer, lab designation VP 2018/14.1, BASF Construction Solutions GmbH, Trostberg, Germany) was added to the suspension. The superplasticizer had a solids content of 23 mass-% and a density of 1.05 g/cm³ [22]. The dosage of the superplasticizer agent (i.e., the polymer) was 0.02 mass-% in relation to cement mass, and it was added 2.5 min after water–cement contact during the mixing process. All cement pastes for rheological investigations were prepared with a standard mixer, as defined in DIN EN 196-1 [23] according to the mixing sequence in Table 1. Masses of 1778.9 g CEM I and 728.0 g water were mixed, resulting in a water/cement mass-ratio of 0.41, corresponding to a volumetric content of solids (in the following designated as phase content) of 0.44. With regard to pastes containing SP, the same amount of cement was mixed with 1.55 g of superplasticizers and 726.5 g of water. The superplasticizer was added according to the timetable given in Table 1. The resulting sample volume was 1.3 dm³ for all prepared pastes.

Following the preparation of the samples, temperature as well as density were determined (not shown). Before measurements, the sealed samples were stored in a tempered water bath at 10, 20 or 30 °C, respectively, in order to keep the sample temperature constant. The cement pastes were investigated for their rheological properties using the procedure described in Section 2.2, starting at 15, 30, 60, 120 and 180 min, respectively, after cement–water contact. Therefore, a sample volume of 301 cm³ was poured into in the rheometer beaker. The rheometer was equipped with a stress-controlled measuring setup. For details on the conversion from the applied torque and rotational speed to the rheological parameters shear stress τ and shear rate $\dot{\gamma }$, see [24]. For additional analysis, a Haegermann slump flow without shocks according to [25] was determined 30 s after the start of each rheological measurement (not shown). A new sample was prepared for each measurement in order to enable an unsheared rest of every sample until measurement.

### 2.2. Methods

#### 2.2.1. Calorimetry

Isothermal conduction calorimetry was performed on cement pastes mixed internally within the calorimeter (ToniCal Trio, Tonitechnik, Berlin, Germany). For each temperature, the calorimeter was calibrated prior to the measurements. From the total released heat, the degree of hydration was calculated under the assumption that the samples hydrated at 30 °C reached complete hydration.

#### 2.2.2. XRD and SEM

X-ray diffraction (XRD) was performed ex situ. For that purpose, approximately 15 g of fresh cement paste hydrated at 10, 20 and 30 °C, respectively, was mixed with 5 mL of 2-propanol to stop hydration after 15, 30, 60, 120 and 180 min of water–cement contact. Subsequently, the liquid phase was removed by drying at 40 °C for 12 hours. XRD-measurements were conducted in a Siemens D 5000 diffractometer (now: Bruker, Karlsruhe, Germany)) using a Cu-tube operating at 40 kV and 40 mA. With a step size of 0.03°, 2Θ the scans were recorded over a range between 6° and 70° 2Θ at an acquisition time of 4 s at each step. The phase quantification was performed using the Rietveld-software Profex (v.3.14.3, Döbelin, Solothurn, Switzerland).

Scanning electron microscopy (SEM) was performed on selected samples already used for XRD. For this purpose, a Nova NanoSEM 230 (FEI, Eindhoven, the Netherlands) equipped with a field emission gun was used.

#### 2.2.3. Ion-Concentration

The cement pastes were centrifuged for each test at 15,000 g (g = 9.81 m/s²) for 8 min. Afterwards, the supernatant solution was removed with a syringe and filtered (syringe filter; 0.45 µm pore size). pH was measured immediately after centrifugation using a glass electrode. For stabilization purposes against precipitation, the aqueous solution was stabilized by addition of 1% HNO_3_ at a dilution factor of 13. The measured concentrations were corrected taking this dilution into account. Ion concentrations were measured by inductively coupled plasma–optical emission spectroscopy (ICP–OES, Horiba, ActivaM, Oberursel, Germany). The degree of supersaturation was calculated from ion activities (Debye-Hückel theory) using the software Phreeqc (cemdata 3T_V07_02 database [26]) and the thermodynamics provided in [27,28,29].

#### 2.2.4. Rheological Measurements

The rheological measurements were performed with a ThermoFisher Haake MARS 60 rheometer (ThermoFisher Scientific, Karlsruhe, Germany). A building-materials cell was used as measuring setup. In this setup, the sample holder is equipped with a tempering unit, keeping the sample temperature during measurement at 10, 20 or 30 °C, respectively. More details about the measuring setup can be found in [3,24].

**Figure 1:**
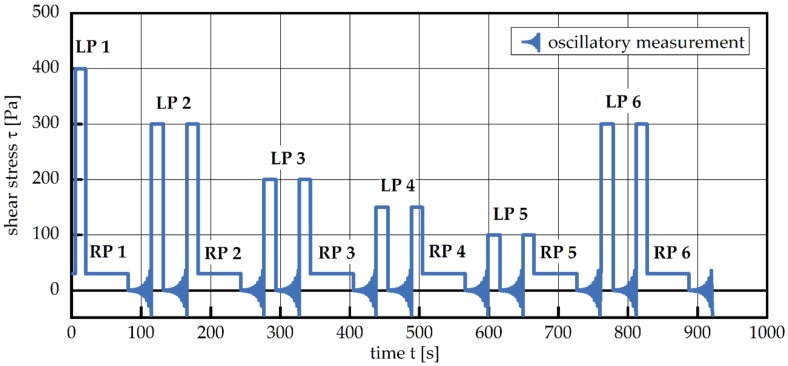
The rheometer measurement sequence consisting of loading phases (LPs) characterized by high stress levels and recovery phases (RPs) with low stress levels. Oscillatory measurements in between loading and recovery phases are employed in order to determine the structural strength; for details see text. The stress value of the oscillatory measurement cannot be quantified in this figure.

The developed measurement sequence consists of a combination of rotational and oscillatory measurements. Successions of sequences with high shear stresses (designated as loading phase, LP) and low shearing stresses (designated as recovery phase, RP) are implemented for analysing the influence of pre-shearing on structural recovery (Figure 1).

**Figure 2:**
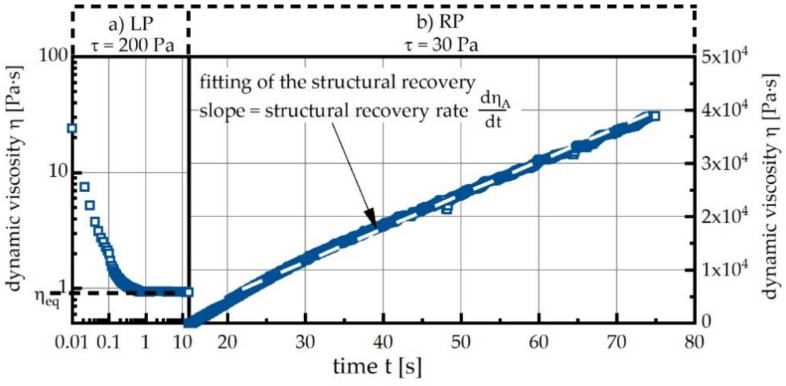
Exemplary depiction of the change in dynamic viscosity observed during the loading phase (LP) and the recovery phase (RP): (**a**) LP causes a non-linear decrease until the equilibrium dynamic viscosity η_eq_ is reached; (**b**) fitting of the linear increase of η during the RP in order to determine the structural recovery rate dη_A_/dt; measurement of a cement paste at 20 °C without superplasticizer starting at an age of 15 min.

Five consecutive loading phases (LPs) with decreasing levels (*τ*_LP1_ = 400 Pa, *τ*_LP2_ = 300 Pa, *τ*_LP3_ = 200 Pa, *τ*_LP4_ = 150 Pa and *τ*_LP5_ = 100 Pa, respectively) are each followed by a recovery phase (RP) with a constant load of *τ*_RP_ = 30 Pa. The load level of LP2 was repeated in LP6 to investigate the influence of the hydration processes during the experiment; i.e., the reversibility. During each loading phase, the structural breakup of particle agglomerates within the cement suspension is studied by withdrawing and shock-freezing samples in liquid nitrogen. As a next step, these samples will be subjected to synchrotron X-ray tomography investigations and will be reported elsewhere. Here, we focus on the change in dynamic viscosity due to the applied shear loading. At constant high shear load, the dynamic viscosity decreases, striving toward a load-dependent plateau value, hereafter referred to as equilibrium dynamic viscosity η_eq_ (Figure 2, LP). A similar procedure is used to describe the following RP. We report the change of dynamic viscosity with time dη_A_/dt, which in all cases exhibited a quasi-linear relationship (Figure 2, RP). This structural recovery rate dη_A_/dt is reported as a function of the shear stress in the preceding LP.

Both after LPs and RPs, the structural limit stress *τ*_s_ [3]—which relates to the yield stress—and the behaviour of the shear modulus |G*| of the suspension were determined by an oscillatory measurement sequence. More specifically, a stress controlled amplitude sweep was carried out, in which the stress amplitude τ_A_ was increased in a stepwise manner, from *τ*_A_ = 0.497 Pa to *τ*_A_ = 485 Pa. In order to prevent excessive shearing of the sample, the amplitude sweep was stopped when the radial deflection *ϕ* of the rotor exceeded 0.1 rad. The measurement frequency was set to *f* = 5 Hz, and the deformation amplitude γ_A_ was recorded. The change of the complex shear modulus |G*| = |*τ*_A_/γ_A_| as a function of *τ*_A_ was evaluated for the stress amplitude *τ*_S_, at which |G*| exhibits a significant drop, using the method described in [3]. This stress is in the following referred to as structural limit stress, *τ*_S_, and relates to the Bingham yield stress of the sample.

## 3. Results

### 3.1. Calorimetry

**Figure 3:**
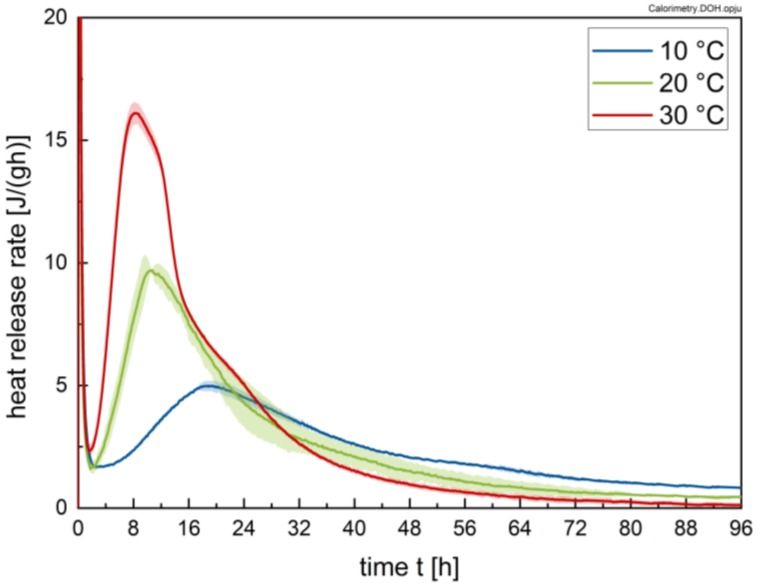
Heat release rate of the cement pastes with w/c = 0.41 without superplasticizer addition at temperatures of 10, 20 and 30 °C, respectively, for a time interval of 96 h.

The heat release rates of the cement pastes for the different temperatures are plotted in Figure 3 as a mean value of three measurements with the respective standard deviations. As expected, the absolute values of the hydration rate increase and the length of the induction period decreases with increasing temperature. The shoulder at the deceleration period is attributed to the sulphate depletion phenomenon, whereby C_3_A hydration is not adequately suppressed [30,31]. Although this effect occurs at hydration stages, which are not relevant for rheology, the results reveal that sulphate depletion depends on temperature, since the associated peak is shifted to later hydration stages with decreasing temperature. Overall, the cement used shows no more than minor sulphate depletion; i.e., optimal sulphatisation.

**Figure 4:**
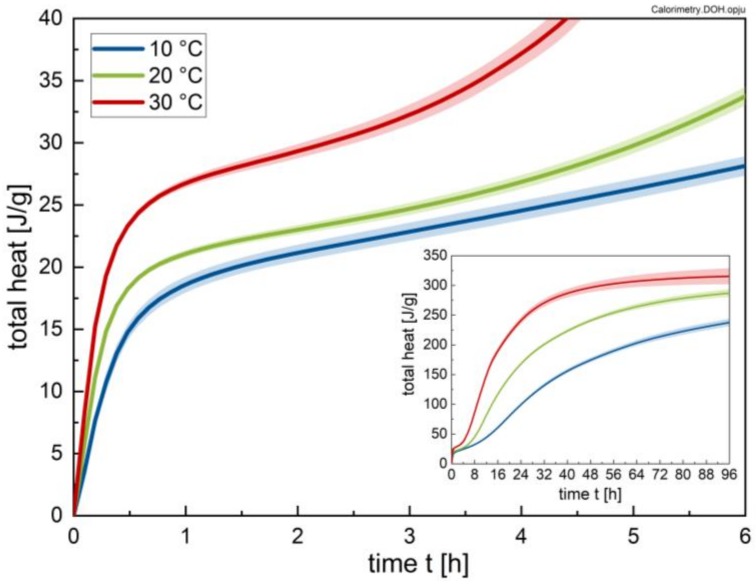
Total heat liberated by the cement pastes (w/c = 0.41; w/o superplasticizer) at temperatures of 10, 20 and 30 °C, respectively, for a time interval of 6 and 96 h.

The total heat, which is released during hydration, increases with increasing temperature, and the time dependence is depicted in Figure 4. The impact of temperature is stronger for the early hydration stage; i.e., the time period up to 48 h. After 96 h of hydration, the difference between 20 °C and 30 °C sample temperature becomes low, but still more heat is liberated in these cases compared to hydration at 10 °C, as seen in the inlet of Figure 4. The total heat after 96 h in the case of the 30 °C samples is used to calculate the degree of hydration assuming that these samples are completely reacted, despite the fact that this values is not reached in practice.

Because the present investigation focuses on the early hydration period, the total heat that is liberated by the cement paste is plotted for the first 6 h of hydration in Figure 4. It can be seen that in the hydration period relevant for the current study, the differences between 10 and 20 °C are small; only the sample hydrated at 30 °C shows an increased release of heat during the first 5 h, indicating an increased hydration progress for this sample compared to the others.

### 3.2. Ion Species in Carrier Liquid and Change in Solid Phase Inventory

**Figure 5:**
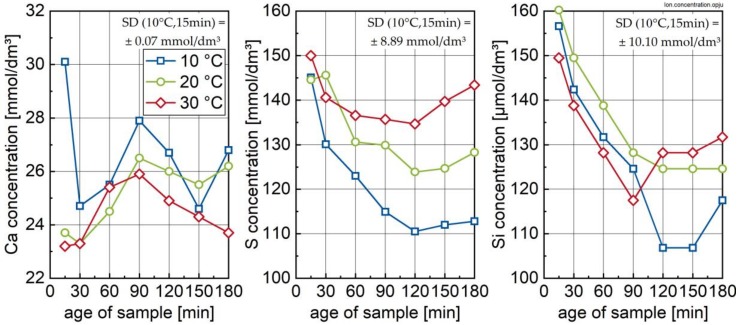
Concentration of the elements Ca (**left**), S (**middle**) and Si (**right**) in the aqueous phase as a function of time after water’s addition to the cement. Standard deviation (SD) calculated from two repetitions.

To get more insight into the cement hydration, the aqueous phase and the solid phase compositions are investigated. The ion concentrations in the aqueous phase during the first 3 h of hydration depending on temperature are reported in Figure 5.

The concentration of Ca ions significantly drops in the time frame between 15 and 30 min after water addition, followed by a temperature-dependent repeated increase in Ca. In contrast, both S (in the present case occurring as SO_4_^2−^) and Si ion concentrations steadily decrease, with sulphur showing clear temperature dependence. The drop in sulphur concentration is interpreted as a clear indication for the formation of ettringite, confirming the results of Jakob et al. [17]. The declining content of Si ions is more difficult to interpret. Based on studies regarding pure C_3_S hydration, the drop in silicon may be attributed to solubility with respect to a precursor phase of mature C-S-H (denoted in the following as metastable C-S-H (C-S-H (m))) [29]. Accordingly, as the calcium ion concentration increases, the silicon concentration decreases due to this equilibrium. Thus, the aqueous phase composition gives clear indication on the formation of metastable C-S-H on the alite surface during cement hydration as well. This observation is in line with actual assumptions on the formation of C-S-H at the beginning of hydration [11,12]. It is reported that metastable C-S-H contains uncondensed silicon tetrahedra (Q^0^), as revealed by various ^29^Si NMR studies [32,33], which is not detectable in XRD.

**Figure 6:**
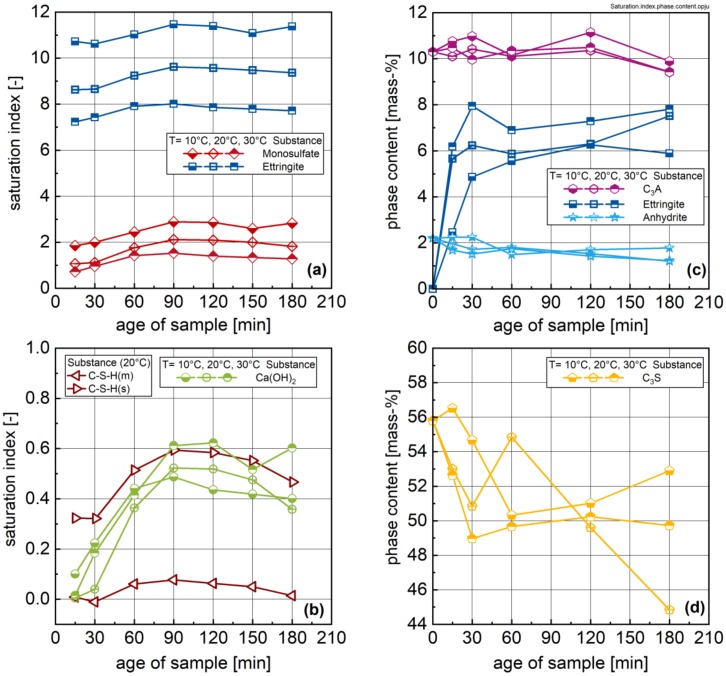
Saturation indices for ettringite and AFm (**a**), Ca(OH)_2_, metastable C–S–H(m) and stable C–S–H(s) (**b**); content of ettringite, C_3_A and anhydrite (**c**); and C_3_S (by QXRD) during the first 3 h of hydration (**d**).

The measured ion concentrations in the aqueous phase were used to calculate saturation indices for ettringite; monosulfate (AFm); bassanite (beta modification); metastable C-S-H (C-S-H(m)) [29] and stable C-S-H (C-S-H(s)) [28]; and portlandite using the software PhreeqC. The results are shown in Figure 6a–c. Additionally, the solid-phase contents determined by quantitative X-ray diffraction (QXRD) are displayed in Figure 6d.

The hydration of the aluminate phases together with the sulphate carrier is discussed first. The calculated saturation indices indicate that the aqueous phase is undersaturated with respect to bassanite (2.7 mass-% in the raw cement; not shown) after only 15 min of hydration. XRD analysis shows that bassanite is not present during further hydration, suggesting that bassanite is completely dissolved during the first minutes of hydration. The QXRD-results indicate that anhydrite dissolves during hydration, but at a lower rate compared to that of bassanite. Significant dissolution of C_3_A cannot be confirmed by QXRD. Dissolution of bassanite and anhydrite together with C_3_A/C_4_AF leads to rapid supersaturation with respect to ettringite, as shown in Figure 6a. After 180 min of hydration, approximately 1 mass-% of C_3_A should have reacted to produce the measured ettringite content. This small change is hard to infer from QXRD. A further source for aluminate ions is C_4_AF. QXRD indicates that more than 1 mass-% C_4_AF dissolves after 15 min of hydration (not shown). Generally, supersaturation with respect to ettringite is higher with lower temperature. This temperature dependency of ettringite saturation is opposed by the trends in the determined ettringite content by QXRD (the standard deviation for ettringite is relatively high); i.e., the ettringite content increases with increasing temperature, suggesting that the ettringite formation is lower at lower temperatures. Comparable results on the ettringite content for the same cement (but lower w/c ratio) are reported by Jakob et al. [17]. Although the aqueous phase is slightly supersaturated with respect to monosulfate (AFm), the presence of AFm could not be confirmed by QXRD. Therefore, it can be concluded that the potential (supersaturation) to form ettringite and monosulfate is given by the ion activities; the formation of these phases occurs slowly.

The hydration of alite is reflected in Figure 6d. The calculated saturation indices show that the aqueous phase is more or less in equilibrium with metastable C-S-H [29] and supersaturated with respect to stable C-S-H ([28]; only the 20 °C samples can be considered due to the lack of the temperature-dependent solubility data for metastable C-S-H). Additionally, a slight supersaturation with respect to portlandite was calculated, with a minor impact from temperature. This supersaturation increases with hydration time until a maximum is reached. Such behaviour is known from investigations of the C_3_S hydration, and the formation of portlandite occurs at very high degrees of supersaturation. Accordingly, it is expected that portlandite is formed after the maximum of its degree of supersaturation (between 90 and 120 min of hydration at 20 °C in the present study). However, the formation of portlandite could not be confirmed by QXRD due to the small phase content and the high uncertainty related to this phase in QXRD. Similarly, the detection of metastable C-S-H (short-range ordered) by XRD is not possible due to low phase concentrations and poor reflections in XRD. However, the amount of alite is reduced during hydration for all temperatures. From the calorimetry data it can be assumed that with increasing temperature, alite dissolution is higher at later hydration periods.

**Figure 7:**
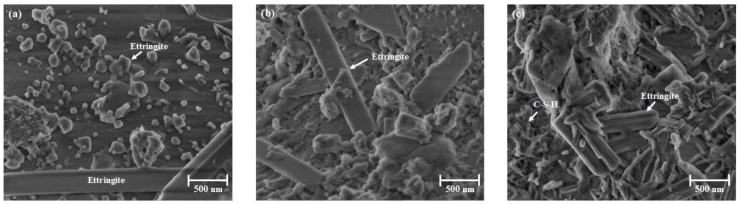
Microstructures of hydrated cement pastes after 15 min at 10 °C (**a**), 20 °C (**b**) and 30 °C (**c**) revealed by SEM.

In order to clarify the role of C-S-H formation, SEM investigations on the microstructures of hydrated cement pastes were conducted. For the sample hydrated for 15 min, Figure 7 shows that small amounts of ettringite are formed at 10 °C. Mostly, the ettringite crystals were small. Only occasionally were large crystals observed. Generally, the cement surface appeared uncovered by hydration products. This is in contrast to the samples hydrated at 20 (Figure 7b) and 30 °C (Figure 7c), for which the surface was rather covered by ettringite with larger individual crystals; i.e., the 30 °C sample showed the highest ettringite content. These observations are in accordance with the findings from the QXRD (see Figure 6). Only in the sample hydrated at 30 °C was the formation of C-S-H and portlandite observed (Figure 7c).

**Figure 8:**
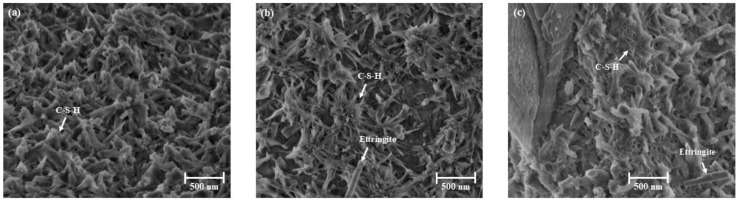
Microstructure of 180 min hydrated cement pastes at 10 °C (**a**), 20 °C (**b**) and 30 °C (**c**) revealed by SEM.

After 180 min of hydration (Figure 8), the microstructure is characterized by a large amount of ettringite visible on the cement surface, and the ettringite appears smaller in crystal size at 10 °C compared to the other temperatures (Figure 8a–c). The images reveal that C-S-H formation is enhanced by increasing temperature (compare Figure 8a and Figure 8b,c). The SEM investigations are again in agreement with XRD.

### 3.3. Change in Rheological Properties

As outlined in Section 1, the primary goal of this study was to investigate the nature of the structure-formation processes taking place in fresh cement pastes at rest and the influence of the shear history and the temperature on these processes.

**Figure 9:**
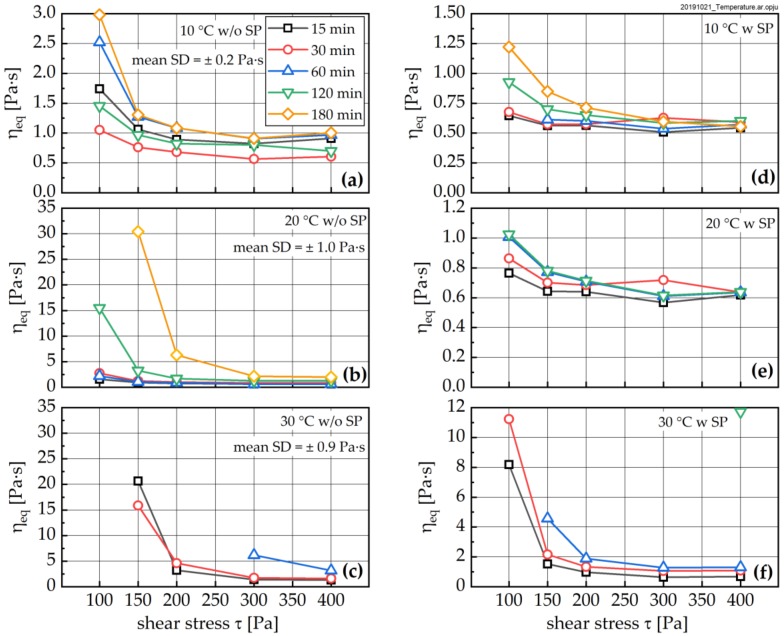
Change of equilibrium viscosity (η_eq_) as a function of applied shear stress for samples without (left; **a**–**c**) and with superplasticizer (right; **d**–**f**) for temperatures of 10 °C (**a**,**d**), 20 °C (**b**,**e**) and 30 °C (**c**,**f**). Note, the 30 °C samples and higher ages values were not measurable, as the samples became too stiff for rheometer measurements. Mean standard deviation (SD) was calculated from three repetitions each time, and all shear stresses at corresponding temperature without SP.

In this context, in a first step, the influence of shear stress during the individual LPs on the equilibrium dynamic viscosity η_eq_ was studied. The dependence of η_eq_ on the applied shear stress is presented in Figure 9 for samples without (left) and with superplasticizer (right). The results show that for a temperature of 20 °C (Figure 9b) and sample ages below approximately 60 min, η_eq_ is quasi-independent of the applied shear stress for stress levels *τ* > approximately 200 Pa, indicating the paste has more or less reached the totally dispersed state. For *τ* < 200 Pa, a pronounced increase in η_eq_ with decreasing shear stress is observed, indicating that the applied stress is not sufficient to totally disperse the agglomerate structure. Figure 9b also shows that the strength of the agglomerate structure is highly dependent on the age of the sample. For ages of 120 and 180 min after water addition, respectively, an increasing influence of the applied shear load *τ* on the equilibrium dynamic viscosity η_eq_ is observed. This indicates that the strength of the agglomerates increases with progressing hydration, and that this increasing change especially occurs for ages greater 1 h (with regard to the 20 °C sample).

The dependencies described above in principle apply to all temperatures. With increasing temperature, a shift of the curves to higher shear stresses is observed, indicating that in order to destroy the paste structure, higher stresses become necessary with increasing temperature. This increase enables drawing the conclusion that the strength of the agglomerate structure is strongly influenced by the hydration of the cement particles.

From Figure 9d–f, showing samples prepared with superplasticizer, it can be seen that the addition of SP reduces η_eq,_ but does not necessarily lead to a shift of the structural strength—i.e., the stress at which no further reduction of the dynamic viscosity can be observed—to lower values. The results rather indicate that SP addition leads to retardation of the hydration, as the increase of η_eq_ with sample age is delayed for samples with SP compared to samples without SP.

**Figure 10:**
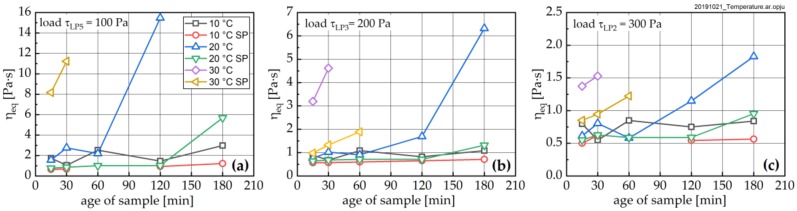
Change of equilibrium dynamic viscosity η_eq_ as a function of time for different temperatures with and without SP addition for shear stresses of 100 Pa (**a**), 200 Pa (**b**) and 300 Pa (**c**), respectively. Mean standard deviation (SD); see Figure 9.

The time-dependence of the results (see Figure 10) suggests that hydration induced effects are especially relevant at low shear stresses and are reduced both with increasing shear stresses and with increasing SP dosage.

**Figure 11:**
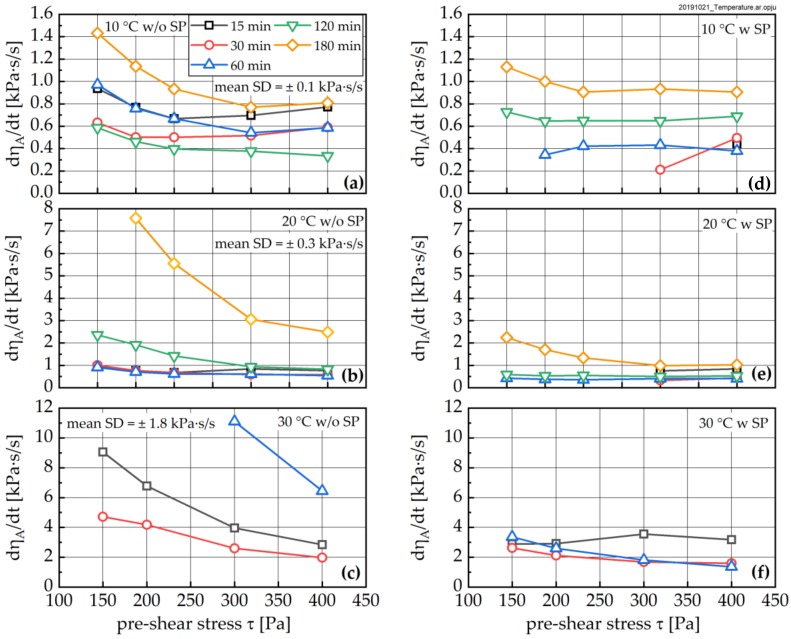
Structural recovery rate dη_A_/dt for pastes without (left, **a**–**c**) and with SP addition (right; **d**–**f**) at different temperatures of 10 °C (**a**,**d**), 20 °C (**b**,**e**) and 30 °C (**c**,**f**). Mean standard deviation (SD) calculated from three repetitions and all shear stresses at corresponding temperature without SP.

Each LP was followed by a structural recovery phase (RP), characterized by constant, low shear stress. Figure 11 shows the structural recovery rate dη_A_/dt as a function of pre-loading stress—i.e., the stress during the LP immediately prior to the RP—and time. As can be seen that with increasing temperature, dη_A_/dt increases significantly. Further, the pastes exhibit a clear memory for the shear history; i.e., for lower pre-shear stresses, all paste show a higher recovery rate. SP addition strongly suppresses the recovery of the paste.

**Figure 12:**
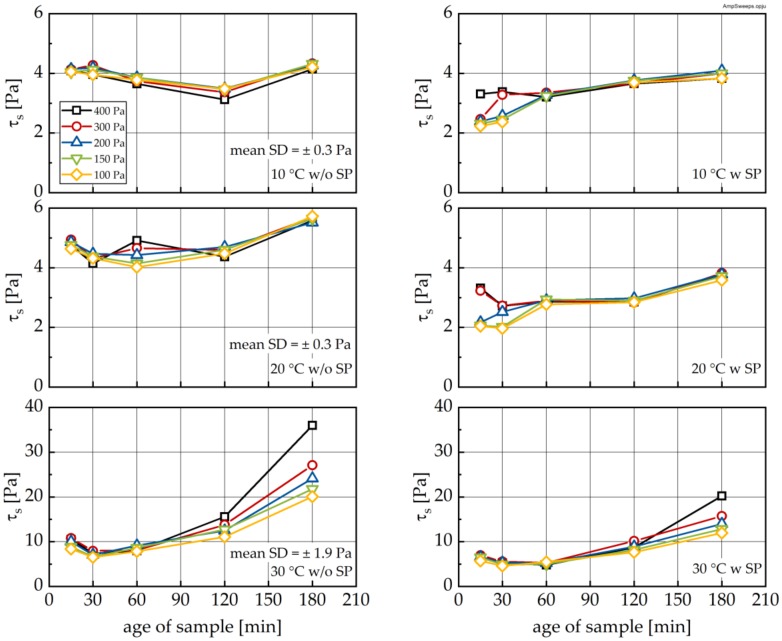
Structural limit stress *τ*_s_ measured after the recovery phase RP as a function of time without (left) and with SP addition (right) for different pre-shear stresses at 10 °C (top), 20 °C (middle) and 30 °C (bottom), respectively. Mean standard deviation (SD) calculated from three repetitions and all shear stresses at corresponding temperature without SP.

Following each high and low shear phase, an oscillatory measurement was performed, in order to quantify *τ*_S_, which is closely related to Bingham yield stress (see [3]). Figure 12 shows *τ*_S_ as a function of sample age and pre-shear stress. As can be seen, in pastes without superplasticizer addition, all curves initially show a decline of the structural limit stress followed by a subsequent increase. These results are largely independent of the pre-shear rate. The minima for *τ*_S_-age curves occur at approximately 120 min for the 10 °C sample, approximately 60 min for the 20 °C sample and approximately 30 min for the 30 °C sample. The addition of SP (see Figure 12, right) shifts the τ_S_ curve to lower values. Interestingly, the previously observed minima seem to disappear.

**Figure 13:**
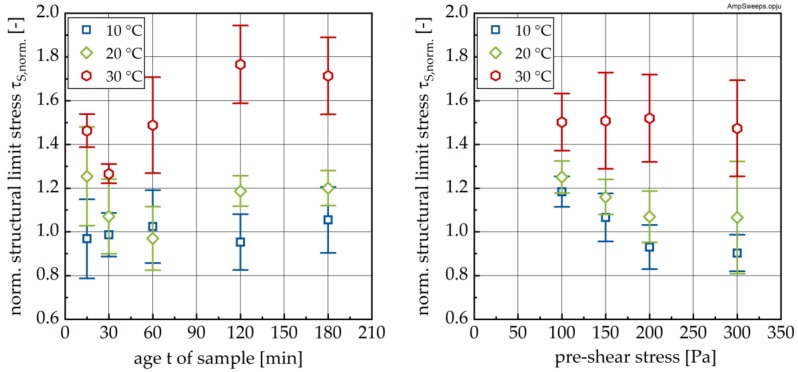
Structural limit stress *τ*_s_ measured after the recovery phase RP normalized by the *τ*_s_ value measured immediately at the end of the loading phase as a function of sample age **(left**) and pre-shear stress (**right**) for different temperatures.

**Figure 14:**
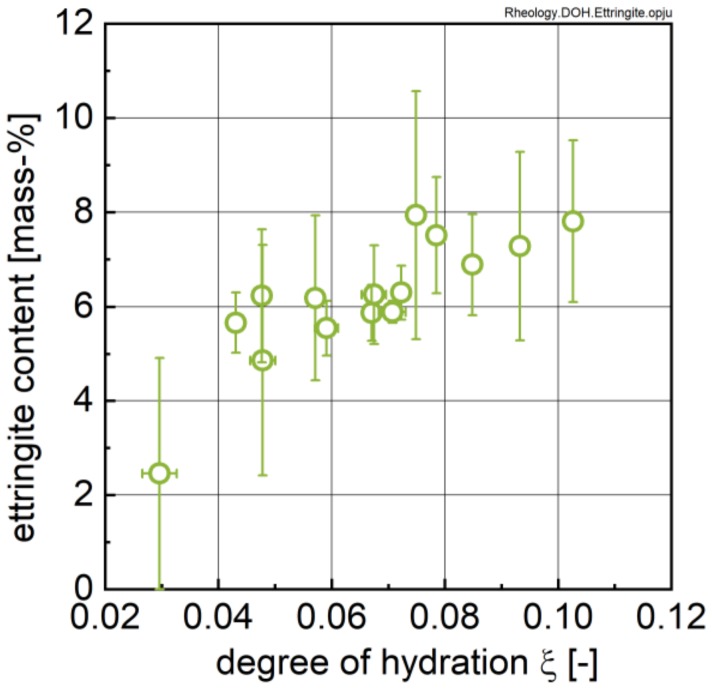
Ettringite content of investigated cement pastes determined via QXRD (see Figure 6) as a function of the degree of hydration calculated from calorimetry (see Figure 4).

In order to evaluate the effect of the RP on *τ*_S_, *τ*_S_ values after RP (compare Figure 12) were normalized to the corresponding values immediately after the end of LP (i.e., before the RP; designated as norm. *τ*_S_; see Figure 13). As can be seen, for the 10 °C sample, the mean τ_S_ does not change significantly during RP (see Figure 13, left). With increasing temperature, a pronounced increase in *τ*_S_ during RP can be observed, reflected by scaled norm. *τ*_S_ values greater than 1.0. Interestingly, recovery shows the same distinct feature of a minimum like that shown in Figure 14.

The effect of the pre-shear stress on structural recovery is shown in Figure 13 (right). Whereas for the 30 °C sample, no influence of the pre-shear stress was observed, increasing pre-shear led to a reduction in the structural buildup for all points in time (see variance).

## 4. Discussion

Using the rheological measurements described in Section 3, the combined effect of temperature and hydration, as well as that of shear on the thixotropy of fresh cement paste, could be quantified. A key question to be discussed in this section is whether the rheological parameters (and their change in time) can be related to cement hydration; i.e., the change in carrier liquid composition and mineral phase (i.e., particle) inventory.

In order to carry out such a comparison, in a first step, ettringite (AFt) formation was compared to an apparent degree of hydration, as calculated from the calorimetric measurements. To this end, the heat evolution at a certain time (see Figure 4) was normalized to the total heat after 96 h of hydration for each temperature. This normalized heat is in the following referred to as degree of hydration.

As can be seen from Figure 14, the ettringite content reaches values between 5.5 and 8.0 mass-% for very low degrees of hydration in accordance to Jakob et al. [17] for the same cement. A linear trend of ettringite content vs. degree of hydration might be inferred, but this does not hold under statistical consideration (Adj. R² = 0.67).

In a next step, the rheological parameters, i.e., the structural limit stress *τ*_s_, the equilibrium dynamic viscosity at defined shear load η_eq_ and the structural recovery rate dη_A_/dt, are compared both to the degree of hydration and the ettringite content in the sample.

**Figure 15:**
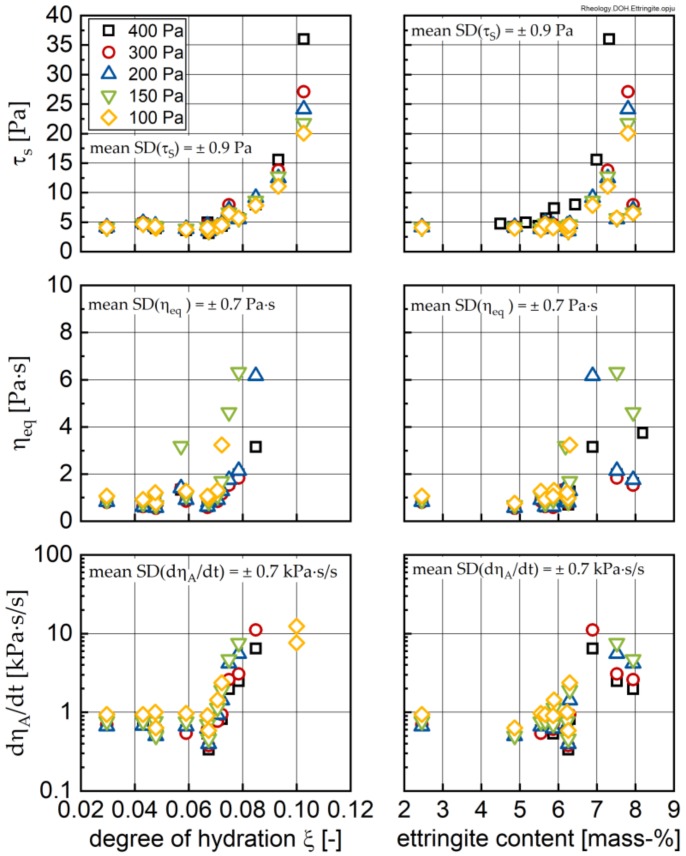
Structural limit stress *τ*_s_, equilibrium dynamic viscosity at defined shear load η_eq_ and the structural recovery rate dη_A_/dt of the investigated cement pastes as a function of the shear stress or pre-shear stress, respectively, plotted over the degree of hydration (**left**; definition see text) and the ettringite content (**right**). Mean standard deviation (SD) calculated from three repetitions for all times, all shear stresses and all temperatures without SP.

In Figure 15 all investigated rheological parameters show an approximated bi-linear trend. Up to a degree of hydration of approximately 0.07 the rheological parameters stay quasi-constant, but with a distinct influence of the applied shear and pre-shear stress being visible. Only for higher degrees of hydration, a significant stiffening of the paste and a pronounced increase in the structural recovery rate can be observed. With regard to the influence of ettringite on the rheological properties, the trend is less clear. Even here, an approximated bi-linear trend can eventually be inferred, with a shift in the influence on rheology occurring at ettringite contents of approximately 6 mass-% to 6.5 mass-%.

**Figure 16:**
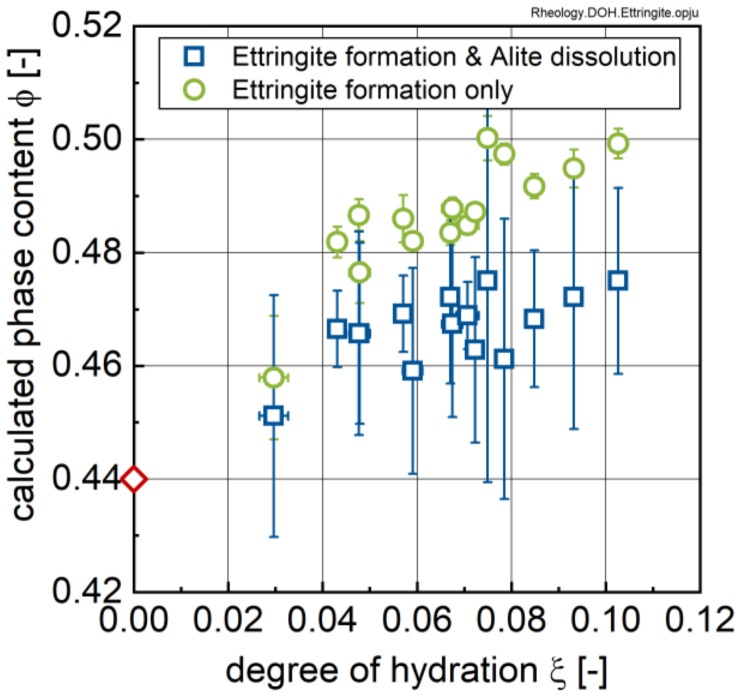
Influence of water absorption due to ettringite formation calculated using Equation (1) and due to combined ettringite formation and alite dissolution calculated from Equation (2), respectively.

This finding seems at first sight to be in contradiction to results by Jakob et al. [17], who observed a continuous increase in the torque moment in rheometer measurements with increasing ettringite content, and thus not the above approximated bi-linear trend. Jakob et al. explained the increase in the rheological properties by a shift in phase content resulting from the water consumption inflicted by the formation of ettringite [17]. Implementing this approach to data from this study yields the results shown in Figure 16. The phase content ϕ, i.e., the volume of solids, *V*_s_, divided by the volume of the of solids, *V*_s_, and liquid, *V*_liq._, was calculated by Equation (1). The volume of the suspension was taken to be 1 cm³ with an initial phase content of ϕ_0_ = 0.44 corresponding to an initial cement volume of 0.44 cm³ (i.e., a cement mass of *z*_0_ = 1.3706 g) and an initial water content of 0.56 cm³ (i.e., a water mass of *w*_0_ = 0.56 g).

(1) $$\phi =V_{s}/\left(V_{s}+V_{liq}\right)$$

(2) $$\begin{array}{c} V_{liq}=V_{liq,0}-V_{liq,AFt\;}=V_{liq,0}-f_{m,AFt}\cdot z_{0}\;\cdot \left(1+f_{m,AFt}+f_{m,CH}\right)\cdot f_{m,H_{2}O},\\ V_{s}=V_{s,0}+V_{s,AFt}\;=V_{s,0}+f_{m,AFt}\cdot z_{0}\;\cdot \left(1+f_{m,AFt}+f_{m,CH}\right)/\varrho_{AFt} \end{array}$$

(3) $$\begin{array}{c} V_{liq}=V_{liq,0}-V_{liq,AFt\;}=V_{liq,0}-f_{m,AFt}\cdot z_{0}\;\cdot \left(1+f_{m,AFt}+f_{m,CH}\right)\cdot f_{m,H_{2}O},\\ V_{s}=V_{s,0}+V_{AFt}-\Delta V_{C_{3}S}-\Delta V_{Anydrite}-\Delta V_{Bassanite}\\ =V_{s,0}+f_{m,AFt}\cdot z_{0}\cdot \frac{\left(1+f_{m,AFt}+f_{m,CH}\right)}{\varrho_{AFt}}-\left(f_{m,C_{3}S}-f_{m,C_{3}S,0}\right)\cdot \frac{z_{0}}{\varrho_{C_{3}S}}\\ -\left(f_{m,Anhydrite}-f_{m,Anhydrite,0}\right)\cdot \frac{z_{0}}{\varrho_{Anhydrite}} \end{array}$$

In Equations (2) and (3), *V*_liq_,_0_ designates the original water content and *V*_liq,AFt_ the liquid bound by the formation of the AFt phase. Accordingly, *V*_s,0_ designates the original solids content, V_AFt_ the amount of AFt phase at a certain point of hydration and Δ*V*_C3S_, Δ*V*_Anhydrite_, Δ*V*_Bassanite_ the volume of C_3_S, anhydrite and bassanite dissolved at the considered point in time. The symbols $f_{m,AFt},$ $f_{m,C3S},$ $f_{m,CH},$ $f_{m,Anhydrite}$ and $f_{m,Bassanite}$ designate the mass fractions of AFt, C_3_S, CH, anhydrite and bassanite on the dry powder after hydration for a certain duration, stoppage with isopropanol and drying, and can be taken from Figure 6. The index 0 designates the respective mass fractions in the dry cement powder (i.e., before water addition). $f_{m,w}$ = 0.459 gives the water content of ettringite by mass from stoichiometry. $\varrho_{AFt}$, $\varrho_{C3S}$, $\varrho_{Anhydrite}$ and $\varrho_{Bassanite}$ designate the densities of the above named phases and were taken to be 1.80, 3.15, 2.97 and 2.70 g/cm³.

As can be seen from Figure 16, the phase content increases significantly with time corresponding to the change in ettringite content (including the observed scatter). In order to explain the approximated bi-linear trend in our data, Equation (1) was amended by the fact that in parallel to the formation of ettringite (which consumes water), the results in Figure 6d prove that alite (C_3_S) is subject to dissolution during this early stage of hydration. Figure 7 and Figure 8 further show that this dissolution does not allow one to detect significant formation of hydration products, i.e., CH and C-S-H, during the early stages of hydration. In Equation (2) the additional effect of alite (C_3_S), anhydrite and bassanite dissolution onto the phase content are considered. Hereby it is assumed that reduction in alite content shown in Figure 6d is caused by dissolution of alite. The authors further assume that at this point no CH and C-S-H precipitation occurs. This assumption certainly has to be questioned, especially for later stages of hydration and for higher temperatures.

The results of this calculation are shown in Figure 16 as well, and clearly indicate that the volume change due to ettringite formation is more or less compensated by the dissolution of alite, which explains the initial constant period in the approximated bi-linear trend observed in Figure 15. Furthermore, it can be expected that other phases (e.g., C_3_A, C_4_AF) and the sulphate carrier dissolve during the early hydration, which reduces, additionally, the solid-phase content in the paste. A key question now is whether the second, increasing period in the trend-lines shown in Figure 15 is caused by ettringite formation, or rather, CH and C-S-H precipitation, or a combination of both. An answer to this question can be found in calorimetry data.

**Figure 17:**
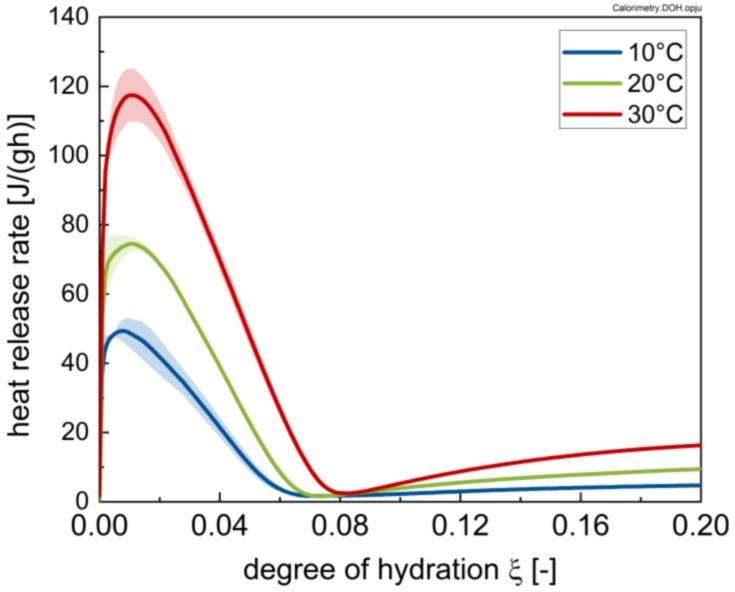
Heat flow of the investigated cement paste for different temperatures (10, 20 and 30 °C) as a function of the degree of hydration.

Figure 17 shows the heat flow as a function of the degree of hydration. The inflexion point in the approximated bi-linear trend observed in Figure 15 (i.e., 0.07) coincides with the degree of hydration where the heat flow curves reach their minimum. The subsequent secondary increase has been clearly identified in the literature as being caused by alite hydration with CH and C-S-H precipitation [30]. Within this context it is suggested that ettringite formation might play a role in the stiffening of the paste, while the main contributions are likely caused by alite hydration (CH and C-S-H formation), in line with previous results [13,18]. This linearity is in line with previous research by the authors [13].

## 5. Conclusions

The goal of the research presented in this paper was to gain a closer understanding of the mechanisms influencing the thixotropy of fresh cement paste and the restructuring of paste at rest especially. Extensive rheological measurements were carried out, in which the yield stress (measured using oscillatory rheology; termed structural limit stress), the dynamic viscosity at high shear loadings and the structural recovery rate at rest were measured at different temperatures. These results were combined with chemical and mineralogical data. The combined experimental results and calculations allow the following conclusions:

- All investigated rheological parameters do not show a continuous change—i.e., increase—with time and degree of hydration, but rather exhibit a steady period or even a minor decline in the first hours of hydration before a pronounced increase is observed. This steadiness or decline cannot be explained by increasing the formation of ettringite. The results, rather, imply that water consumption due to ettringite formation is compensated by the dissolution of alite and other cement compounds, eventually leading to even lower phase contents and thus lower rheological values with time compared to the time of the first measurement; i.e., 15 min.
- The rheological properties show an approximated bi-linear dependency on the degree of hydration. For all measured samples and temperatures, the rheological parameters remain constant or show the previously described minimum up to apparent degrees of hydration of approximately 0.07. Beyond this value, a pronounced increase in the rheological parameters can be observed. From a comparison with the calorimetric data, this increase can be clearly linked to the beginning of the main period of alite hydration with the precipitation of CH and C-S-H. A contribution from ettringite to the change in rheology is likely, but cannot be considered as a major driving force for the stiffening of the paste.
- Fresh cement paste is highly sensitive to the shear history of a given sample. The higher the shear stress the sample was previously exposed to, the lower the structural recovery rate. It should be noted that this relation becomes extremely important for apparent degrees of hydration beyond 0.07, but it is also visible for younger samples. The applied shear stress even has a pronounced influence on the equilibrium dynamic viscosity, which—as expected—decreased with increasing stress. In contrast, the structural limit stress seems to be unaffected by the shear history of the sample for early ages, and only for the 30 °C sample was an effect observed.

## Figures and Tables

**Table 1:** Mixing sequence for sample preparation with a mixer according to DIN EN 196-1 [23].

Procedure	Mixing Intensity	Duration (s)
Homogenization of dry mixture	Level 1	60
Addition of water and mixing process	Level 1	60
Rest phase and manual return of caking material	–	90
Addition of SP and mixing process	Level 2	60
Rest phase and manual return of caking material	–	30
Mixing process	Level 2	120

## References

[1] Banfill P.F.G. (2006). Rheology of fresh cement and concrete. Rheology Reviews 2006.

[2] Verwey E.J.W., Overbeek J.T.H.G. (1948). Theory of the Stability of Lyophobic Colloids.

[3] Haist M. (2010). Zur Rheologie und den physikalischen Wechselwirkungen bei Zementsuspensionen.

[4] Lowke D. (2013). Sedimentationsverhalten und Robustheit Selbstverdichtender Betone—Optimierung auf Basis der Modellierung der interpartikulären Wechselwirkungen in zementbasierten Suspensionen. Ph.D. Thesis.

[5] Wallevik J.E. (2003). Rheology of particle suspensions: Fresh concrete, mortar and cement paste with various types of lignosulfonates. Ph.D. Thesis.

[6] Powers T.C. (1969). The Properties of Fresh Concrete.

[7] Lura P., Friedemann K., Stallmach F., Mönnig S., Wyrzykowski M., Sarmento Esteves L.P., Mechtcherine V., Reinhardt H.M. (2012). Kinetics of Water Migration in Cement-Based Systems Containing Superabsobent Polymers. Application of Superabsorbent Polymers (SAP) in Concrete Construction: RILEM State of the Art Reports.

[8] Yang M., Neubauer C.M., Jennings H.M. (1997). Interparticle potential and sedimentation behavior of cement suspensions: Review and results from paste. Adv. Cem. Based Mater..

[9] Flatt R.J. (2004). Dispersion forces in cement suspensions. Cem. Concr. Res..

[10] Flatt R.J., Bowen P., Siebold A., Houst Y.F. Cement Model Powder for Superplasticizer Properties Studies. Proceedings of the 11th International Conference on the Chemistry of Cement.

[11] Roussel N. (2006). A thixotropy model for fresh fluid concretes: Theory, validation and applications. Cem. Concr. Res..

[12] Roussel N., Ovarlez G., Garrault S., Brumaud C. (2012). The origins of thixotropy of fresh cement pastes. Cem. Concr. Res..

[13] Bogner A., Link J., Baum M., Mahlbacher M., Gil-Diaz T., Lützenkirchen J., Sowoidnich T., Heberling F., Schäfer T., Ludwig H.M. (2020). Early hydration and microstructure formation of Portland cement paste studied by oscillation rheology, isothermal calorimetry, 1H NMR relaxometry, conductance and SAXS. Cem. Concr. Res..

[14] Gebauer J. (1978). Technological possibilities of avoiding the early setting of cement. Zement-Kalk-Gips.

[15] Nonat A., Mutin J.C., Lecoq X., Jiang S.P. (1997). Physico-chemical parameters determining hydration and particle interactions during the setting of silicate cements. Solid State Ionics.

[16] Barnes P., Bensted J. (2002). Structure and Performance of Cements.

[17] Jakob C., Jansen D., Ukrainczyk N., Koenders E., Pott U., Stephan D., Neubauer J. (2019). Relating ettringite formation and rheological changes during the initial cement hydration: A comparative study applying XRD analysis, rheological measurements and modelling. Materials.

[18] Uchikawa H., Ogawa K., Uchida S. (1985). Influence of character of clinker on the early hydration process and rheological property of cement paste. Cem. Concr. Res..

[19] Rößler C., Eberhardt A., Kučerová H., Möser B. (2008). Influence of hydration on the fluidity of normal Portland cement pastes. Cem. Concr. Res..

[20] German Institute for Standardization (2011). DIN EN 197-1: Cement, Part 1, Composition, Specifications and Conformity Criteria for Common Cement.

[21] Lu Z.C., Haist M., Ivanov D., Jakob C., Jansen D., Leinitz S., Link J., Mechtcherine V., Neubauer J., Plank J. (2019). Characterization data of reference cement CEM I 42.5 R used for priority program DFG SPP 2005 “Opus Fluidum Futurum—Rheology of reactive, multiscale, multiphase construction materials”. Data Brief.

[22] DIN German Institute for Standardization (2016). DIN EN ISO 2811-1: Paints and Varnishes—Determination of Density—Part 1: Pycnometer Method.

[23] DIN German Institute for Standardization (2016). DIN EN 196-1: Methods of Testing Cement—Part 1: Determination of Strength.

[24] Haist M., Link J., Nicia D., Leinitz S., Baumert C., von Bronk T., Cotardo D., Pirharati M.E., Fataei S., Garrecht H. (2020). Interlaboratory study on rheological properties of cement pastes and reference substances—Comparability of measurements performed with different rheometers and measurement geometries. Mater. Struct..

[25] DIN German Institute for Standardization (1999). DIN EN 1015-3: Methods for Test for Mortar for Masonry—Part 3: Determination of Consistance of Fresh Mortar (by Flow Table).

[26] Lothenbach B., Kulik D.A., Matschei T., Balonis M., Baquerizo L., Dilnesa B., Miron G.D., Myers R.J. (2019). Cemdata18: A chemical thermodynamic database for hydrated Portland cements and alkali-activated materials. Cem. Concr. Res..

[27] Lothenbach B., Matschei T., Möschner G., Glasser F.P. (2008). Thermodynamic modelling of the effect of temperature on the hydration and porosity of Portland cement. Cem. Concr. Res..

[28] Bullard J.W., Scherer G.W. (2016). An Ideal Solid Solution Model for C–S–H. J. Am. Ceram. Soc..

[29] Sowoidnich T., Bellmann F., Damidot D., Ludwig H.-M. (2019). New insights into tricalcium silicate hydration in paste. J. Am. Ceram. Soc..

[30] Taylor H.F.W. (1997). Cement Chemistry.

[31] Lerch W. (1946). The influence of gypsum on the hydration and properties of Portland cement pastes. Am. Soc. Test. Mater..

[32] Rodger S.A., Groves G.W., Clayden N.J., Dobson C.M. (2005). Hydration of Tricalcium Silicate Followed by 29Si NMR with Cross-Polarization. J. Am. Ceram. Soc..

[33] Bellmann F., Damidot D., Möser B., Skibsted J. (2010). Improved evidence for the existence of an intermediate phase during hydration of tricalcium silicate. Cem. Concr. Res..

